# Actors, technology and geography: arenas of political economy for industrial decarbonisation policy in the United Kingdom

**DOI:** 10.1038/s44432-026-00022-4

**Published:** 2026-11-04

**Authors:** Benjamin K. Sovacool, Matthew Lockwood, Marc Hudson, Kyle S. Herman, Marfuga Iskandarova

**Affiliations:** 1 Boston University, Boston, MA USA; 2 Bennett Institute for Innovation and Policy Acceleration, University of Sussex, Falmer, Brighton, UK; 3 Luiss Guido Carli, Viale Romania, Roma, Italy

**Keywords:** Environmental social sciences, Politics and international relations

## Abstract

Industrial decarbonization (ID) involves significant uncertainty about technical and economic pathways, and it interacts with industrial and trade policy. The UK has been an early leader in ID efforts, developing a specific strategy and a set of deployment policies in the early 2020 s, with a strong focus on carbon capture and storage (CCS) and hydrogen, and a regional ‘cluster’ approach. In this article, we assess the policy choices the UK has made, through the application of a novel framework for analyzing their ‘arenas of political economy’. This framework incorporates three arenas of potential conflict: one over the distribution of policy costs and benefits across actors, a second over the distribution of policy resources between decarbonization solutions or technologies, and a third over the geographical distribution of benefits across industries and the spatial politics of regions. We show that the UK has developed a strong industrial decarbonization policy despite weak industrial policy and an unsupportive institutional context, driven by a stronger advocacy coalition and attempts at redistributing spatial resources to level up deprived regions. However, ID policy also risks being over-complex, being captured by particular technology advocacy groups, and losing credibility and support by concentrating resources too narrowly in particular regions.

## Introduction

Industrial production is responsible for approximately 33% of carbon dioxide emissions globally and represents the fastest growing source of global emissions^1^. Until recently, emissions from energy intensive industries (EII) received little attention, seen as a ‘hard-to-abate’ sector relative to others such as electricity generation. However, following the UNFCCC Paris Agreement in 2015 and the adoption of net zero targets in many countries, pressure to decarbonise industry increased rapidly. Major economies adopted new regulatory frameworks such as the EU’s Clean Industrial Deal, and the Inflation Reduction Act in the USA, which was also developed as a response to the global Covid-19 pandemic and increasing geopolitical pressure from China.

The industrial decarbonisation (ID) challenge is complicated because it covers a range of industries (iron & steel, cement, refining, chemicals, glass, ceramics, pulp and paper, food and drink etc.), and multiple processes that produce GHG emissions from energy use, especially heat, and directly from industrial processes^2,3^. There is also a wide range of potential technical solutions that differ across these industries, both on the supply side and on the demand side^4,5^.

The challenge is heightened by the fact that many of the industries involved make traded products that face sharp competition internationally. At the same time, decarbonising industry will require significant investment; for example, one recent estimate for the UK is more than £500 billion is needed to 2050^6^. This is likely to not only increase product costs, but also risk carbon leakage^7^, leading to calls for policies such as product standards and carbon border taxes in order to avoid carbon lock-in^8^. This international competitiveness dimension of ID also means that it interacts closely with existing industrial policy.

The UK has emerged as an early leader in ID policy^9–11^. It is one of the few countries to have a specific ID strategy document with associated targets^12^, developing a sophisticated set of support policies focusing on carbon capture and storage (CCS) and hydrogen, and seeking to deploy support in ‘clusters’ with concentrations of high emitting industries^13–15^. We ask: how has this specific ID policy pathway emerged, and how does it redistribute resources across differing actors, technology sectors, and geographic places?

To address this question, we identify three ‘arenas of the political economy’ of ID policy, all involving potential conflicts over the allocation of costs and benefits of such policy: i.e., between actors, concerning technology, and across geographic space and regions. These arenas are interrelated and interact with the political economy of wider industrial policy and of regional inequality. These conflicts also carry risks for ID policy itself, ranging from complexity in policy design to capture by technology advocacy groups and disengagement by excluded regions^16^ or even the “waterbedding” of risks to other sectors or locations^17^.

In doing so, the paper addresses a theoretical gap in the growing literature on ID. While recent work maps out approaches for understanding ID at a high level^18,19^, a more developed and nuanced political economy approach is missing, as well as one emphasizing tensions and risks. The paper provides a novel synthesis of political economy theories in an emerging area of climate policy. We agree with Da Costa Vieira^20^, who writes that we need more refined empirical examination of policymakers and how they navigate the contradictions of environmental policy, as well as with Newell and Daley^21^, who argue that we need a better understanding of strategies of alliance-building and counter-pressure related to net-zero policy implementation. All these themes are evident in our paper.

A second contribution is empirical, i.e., a detailed study of the political economy of ID policy in the UK, complementing recent studies that take a more organisational perspective^22^. Upham et al.^23^ apply a ‘just transitions’ lens to the UK case, but they focus on issues of governance and fairness *within* clusters rather than wider distributional aspects across space and time. The UK case study is based on a range of different sources, including policy documentation, industry and NGO reports, electoral data and a set of over 100 interviews with observers of and participants in ID policy processes. More details on the interviews are given in Methods and Supplementary Information.

## Results

### Analytical approach: “arenas of political economy”

In many countries, including the UK, ID has not been politically polarised in a conventional, partisan sense^24–26^. Instead, we argue here that the political economy of ID policy involves a framework we term “arenas of political economy.” Rooted in the comparative political economy, public policy, political science, global governance, and development studies literatures, arenas of political economy refer to how people and organizations function within different contexts, and how these interactions can shape policy outcomes^27^. Arenas may be both formal, such as parliament or a legislature, or informal, such as the rules and norms adopted by community groups, firms, or voluntary associations^28^. When applied to ID, arenas of political economy also include potential or actual conflicts about the distribution of costs and benefits .

Here, we identify three distinct arenas, inspired by Hochstetler’s^29^ concept of “arenas” in the political economy of renewables, although we have adapted this framework to the ID case. The three arenas we identify are distribution of costs and benefits across actors (industry, taxpayers and consumers); technologies (particular decarbonization solutions); and geography (spatial regions and their distribution of industries).

The first of these involves conflict over the *distribution of policy costs and benefits across industry, taxpayers and consumers*, which determines the total amount of resources available for ID. Decarbonising industries will require significant investment, but they are also vulnerable to carbon leakage, so some form of subsidy and/or protection will be needed. This will have to be financed via transfers from taxpayers and/or consumers to EIIs. In this sense, the political economy of ID policy is similar to that of industrial policies aimed at promoting international competitiveness, that have reappeared in the wake of the global financial crisis^30^. The decarbonisation of EIIs sits on top of and interacts with those policies.

The comparative political economy literature suggests that the UK’s institutions should not be conducive for either industrial policy or support for ID. First, we would expect higher levels of support in countries where EII interests are more powerful, i.e., where they play a relatively large role in the economy. By contrast, the UK’s strong neo-liberal economic policy paradigm^31^ facilitated rapid and extensive deindustrialisation in the 1980s and 1990s, and also witnessed the dismantling of trade unions. It is regarded as now having a consumption-led growth model in which services rather than manufacturing are core to the economy^32^.

Second, we would expect support for industry to be greater in countries with corporatist institutions, i.e., where industry has privileged, institutionalised access to policymakers^33^. Taxpayers and/or other consumers, i.e. small firms and households, have to bear the greater costs of this support, but this is argued to be politically feasible because countries with corporatism also tend to have electoral systems with proportional representation (PR), which decreases electoral competitiveness^34^. By contrast, the UK is typically seen as having pluralist institutions with low levels of industry coordination. It also has a first-past-the-post electoral system, two historically dominant political parties and high levels of political competitiveness^35^.

Consumer and taxpayer interests are often seen as being represented within government by finance ministries, while industry interests are seen as being represented by business departments. We would therefore expect to see conflicts between these interests played out through bureaucratic politics. The finance ministry in the UK, i.e. HM Treasury, is seen as being particularly powerful, enforcing a commitment to free markets and control over spending^36^ and expressing scepticism about industrial policy^37,38^.

However, a different view is that, while institutions matter, contingent political factors also play a role in the willingness of the state to redistribute to industry. For example, Craig^39^ argues that the power of the Treasury to assert control over policy ebbs and flows with ‘the ability of forces within and beyond the state to resist its priorities’. Craig also identifies ‘a long-standing tendency for the Treasury to assert a position of power vis-à-vis departmental rivals in ways that undermine their capacity to conduct interventionist industrial policies.’ This points to the possibility that contingent political factors may sometimes overcome or change tendencies generated by institutions, and that we would expect to see such dynamics in conflicts over resources for ID.

**Fig. 1:**
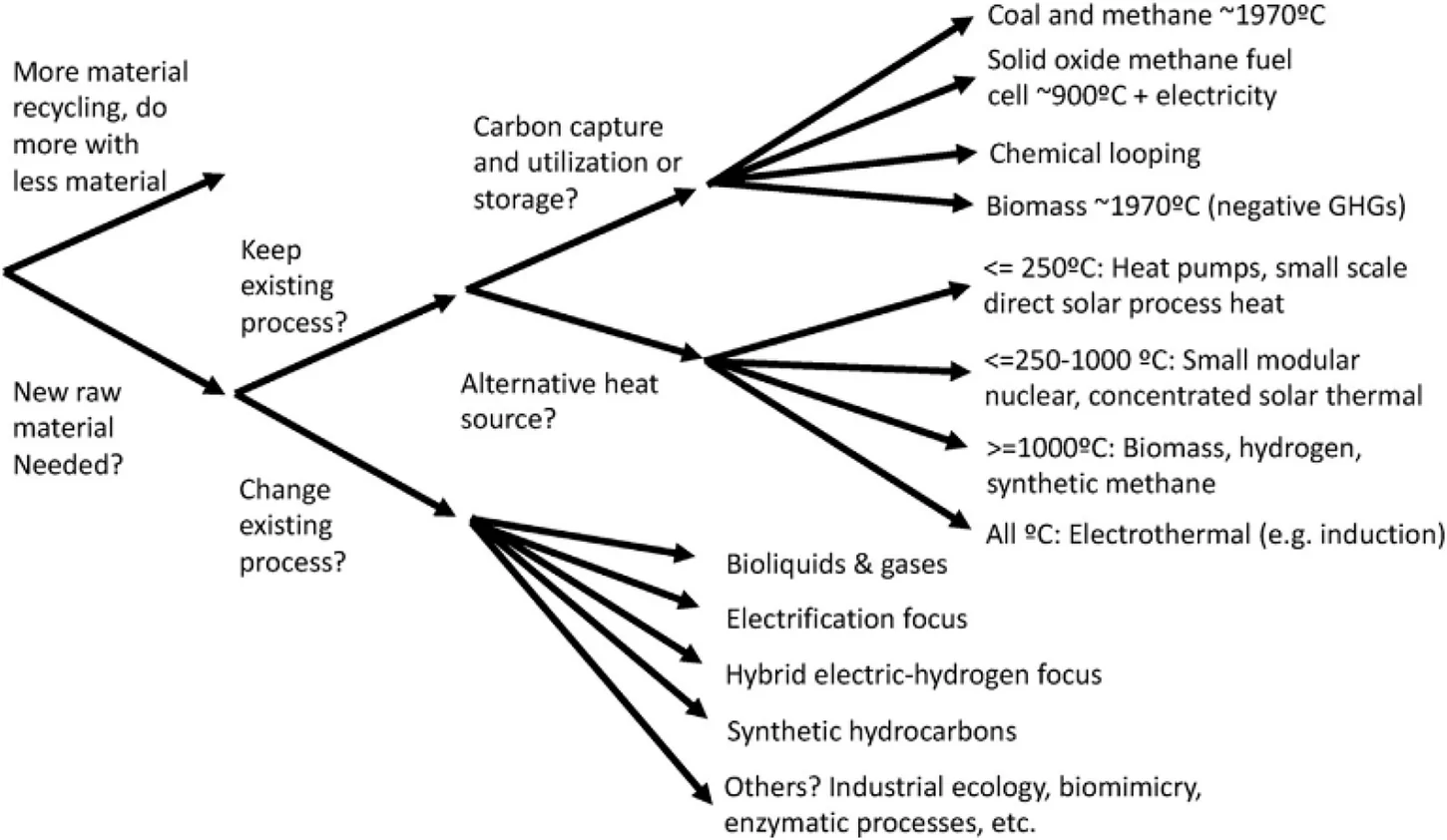
Industrial decarbonisation technology options. Source^4^: The diagram shows a diversified portfolio of decarbonization options that branch from key decision-making criteria such as the use of new materials or recycled materials, heat sources, and processes.

Once these resources have been determined at the macro-level, a second arena involves the *distribution of policy benefits across different types of technology and decarbonisation solutions*. Industrial production typically involves multiple products and processes^40–43^. At the same time, many of these processes may have more than one technically feasible decarbonisation option (Fig. 1).

These technical options for ID include demand side approaches such as recycling or reusing materials, reducing demand through substitution (e.g., timber instead of steel in construction) or by increasing material efficiency in end use^44^. On the supply side, options include changing industrial processes, switching from fossil fuels to low-carbon heat sources, e.g. low-carbon electricity or hydrogen^45,46^, or capturing carbon^47^. There are currently some technical constraints for some options; for example, the electrification of heat is easier for lower temperature processes, and process emissions imply that carbon capture is necessary for decarbonising cement production. But even in these areas there is potential for innovation to reduce or eliminate these constraints^48,49^.

Some solutions such as hydrogen-fired electricity generation or bioenergy with carbon capture and storage, while technically feasible in principle, are not mature^50^—and both industry and governments face uncertainty about which routes to decarbonisation will eventually become the most competitive. While this uncertainty creates a rationale for innovation policy, it also means there are incentives and opportunities for lobbying to secure support for particular technologies and solutions, thereby gaining an early advantage over competitors^51^.

The literature on technology lobbying suggests the importance of coalitions^52–54^. We might expect actors in incumbent decarbonisation technologies, such as natural gas operators supporting blue hydrogen applications, to already be in dominant coalitions. We might also expect more coalitions for supply side decarbonisation solutions rather than demand side solutions. The former typically have a clear focus of common interest^55^, such as a technology or product. They also offer EIIs (and unions and the government) the prospect of maintaining or expanding industrial production. Conversely, on the demand side there are typically many actors across a range of sectors (construction, automotive, etc.) and a politically less attractive message that decarbonisation will be achieved through reduced industrial output. However, at the same time, the more successful the lobby, the more likely it is to provoke counter-lobbies.

The third arena involves *the distribution of policy benefits across geographical regions and industries.* The geographical location of industries has historically depended on a variety of factors, including sources of raw materials and energy, ports, railways and other transport infrastructure, core markets etc., as well as agglomeration effects, creating path-dependent geographies of industrialisation^56,57^. Resulting patterns can vary significantly^58^. For example, cement production in the UK is quite dispersed^59^.

This history means that deindustrialisation in advanced capitalist economies has also been geographically uneven and often concentrated, with lasting negative impacts on employment, incomes and wider deprivation in industrial regions^60–62^. In this context, ID policy offers what remains of EIIs and their regions the prospect of future survival and possibly re-expansion. Retaining manufacturing in such regions is important because it produces jobs with relatively high productivity and wages, and because it can attract foreign investment.

The nature of the regional and industrial politics of decarbonisation policy will depend on the policy approach taken. In the UK case, where policy has concentrated resources on some regions and not others, benefits will inevitably be unevenly distributed, at least initially. As a result, we might expect to see tensions between regions, or between regions and central government. At the same time, industries may be unevenly distributed across clusters, so such tensions may also play out within an industry, with some firms benefitting while others do not, leading such industries to lobby for changes in the approach.

In the next sections, we will briefly discuss the evolution and history of UK ID policy before moving into the core qualitative insights gleaned from our data. We will return to the three arenas of political economy later in the paper.

### Background and context: the evolution of UK industrial decarbonisation policy

Before we jump into our main insights, it is helpful to first provide some context. ID began to come on to the policy agenda in the UK in a significant way in 2017 with the development of the *Industrial Strategy*^63^, closely followed by a *Clean Growth Strategy*^64^. These strategies signalled a commitment to CCS as an important element^65^, which was then reinforced in a 2018 CCS action plan that contained a target for the development of a first CCS plant by the mid-2020s.

In 2019, the UK legislated for a 2050 net zero target, which pointed to the need for accelerated action on ID. This was followed in 2020 with a *Ten Point Plan for a Green Industrial Revolution*, which included targets for low-carbon hydrogen production (capacity of 5 GW of low-carbon hydrogen by 2030) and for the deployment of CCS in two industrial clusters by 2025 and in four clusters by 2030.

The main *Industrial Decarbonisation Strategy* appeared in 2021, laying out a target for emissions reduction from industry of 3 MtCO_2_e emissions per year by 2030. It also formalised the cluster approach with targets of developing at least four low-carbon clusters by 2030, and at least one net zero cluster by 2040. Along with CCS, the strategy highlighted fuel switching away from gas and coal to low-carbon fuels such as electricity, hydrogen, and bioenergy, with a target of 20 TWh.

Shortly afterwards, the government also published a *Net Zero Strategy*^66^, which included a target of carbon capture of between 20 and 30 MtCO_2_e by 2030. The subsequent *Powering Up Britain* energy strategy^67^ increased this to around 50 MtCO_2_e by the mid-2030s. A separate *UK Hydrogen Strategy*^68^ was also published repeating the 5 GW by 2030 target for low carbon hydrogen, but this was then doubled in the 2022 *British Energy Security Strategy*^69^ to 10 GW, with at least half in the form of electrolytic (‘green’) hydrogen. A summary of relevant targets for ID is given in Table 1.

**Table 1:** Targets in UK industrial decarbonisation policy

Area	Target	Date
Industrial emissions reduction	3 MtCO_2_e/year	2030
CCS	20-30 MtCO_2_e captured per year	2030
	first CCS plant	Mid-2020s
Hydrogen production capacity	10 GW (at least half to be electrolytic)	2030
Fuel-switching	20 TWh of fossil fuel use displaced by low-carbon alternatives	2030
Clusters	At least four low-carbon clusters	2030
	At least one net zero cluster	2040

To meet these goals, a number of policy instruments were signalled in the *ID Strategy* and developed over the next two years (Table 2). For CCS, £1 billion was earmarked for development and capital expenditure support. In addition, there was a 15-year revenue support mechanism (or ‘business model’) for projects involving carbon capture. This support bridges the gap between the expected cost of carbon capture and a reference price tied to the UK ETS carbon price. The strategy also proposed a transport and storage regulated investment (TRI) business model which will allow pipeline and storage operators to charge industrial emitters for use of the CO_2_ network. A similar set of proposals for capital and revenue support were made for hydrogen, the latter bridging the gap between the cost of low-carbon hydrogen and that of natural gas. A TRI business model for hydrogen pipeline and storage operators was also proposed.

**Table 2:** UK Government instruments and funding for industrial decarbonisation

Area	Instrument	Date	Funding
CCS	Carbon Capture and Storage (CCS) Infrastructure Fund	2020 onwards	£1bn^a^
	CCUS Business models	2023 onwards	Tbd, but the current government pledged £21.7 billion over 25 years^b^
	CCUS innovation 2.0 programme	2022	£20m^c^
Hydrogen	Hydrogen Business models	2025?	Tbd, estimated at £3.5bn/year by 2030
	Net Zero Hydrogen Fund (NZHF)	2022 onwards	£240m^d^
	Low Carbon Hydrogen Supply Competition	2018 onwards	£33m^e^
Energy efficiency	Industrial Energy Efficiency Accelerator	2017-2025	£24.6m^f^
	Industrial Energy Transformation Fund	2020 - 2028	£500 m^g^
	Industrial Heat Recovery Support	2018 - 2022	£18m^h^
R&D/ Innovation	Clean Growth Equity Fund	2022 onwards	£20 m^i^
	Clean Steel Fund	?	£250m^j^
	Industrial Strategy Challenge Fund (ISCF)	2019-2023	£170 m
	Transforming Foundation Industries	2020-2024	£66m^k^
Supply chains	Green Industries Growth Accelerator (GIGA)	2024 onwards	£960 m^l^
Trade	Carbon Border Adjustment Mechanism	Under consultation, to be implemented 2027	N/A

a: https://www.gov.uk/government/publications/carbon-capture-usage-and-storage-net-zero-investment-roadmap/ccus-net-zero-investment-roadmap-capturing-carbon-and-a-global-opportunity.

b: https://www.gov.uk/government/news/government-reignites-industrial-heartlands-10-days-out-from-the-international-investment-summit.

c: https://www.gov.uk/government/collections/carbon-capture-usage-and-storage-ccus-innovation-20-programme.

d: https://www.gov.uk/government/publications/net-zero-hydrogen-fund-strand-1-and-strand-2.

e: https://www.gov.uk/government/publications/hydrogen-supply-competition.

f: https://www.gov.uk/government/publications/industrial-energy-efficiency-accelerator-ieea.

g: https://www.gov.uk/government/collections/industrial-energy-transformation-fund.

h: https://www.gov.uk/guidance/industrial-heat-recovery-support-programme-how-to-apply.

i: https://www.gov.uk/government/publications/clean-growth-equity-fund.

j: https://www.gov.uk/government/calls-for-evidence/creating-a-clean-steel-fund-call-for-evidence.

k: https://www.ukri.org/what-we-do/browse-our-areas-of-investment-and-support/transforming-foundation-industries/.

l: https://www.gov.uk/government/calls-for-evidence/green-industries-growth-accelerator-hydrogen-and-ccus-supply-chain.

The strategy also included smaller scale instruments to support industrial energy efficiency, research and innovation, and supply chain development. Finally, it discussed a set of related policy challenges including preventing carbon leakage (including by carbon border adjustment mechanisms and/or product standards) and training workers with new skills for decarbonised industries. To deliver this agenda, the team leading ID policy in the energy department (BEIS, then, now the Department for Energy Security and Net Zero (DESNZ) from early 2023) was expanded from a handful in the late 2010s to well over a hundred by the early 2020 s (Interview R33).

**Fig. 2:**
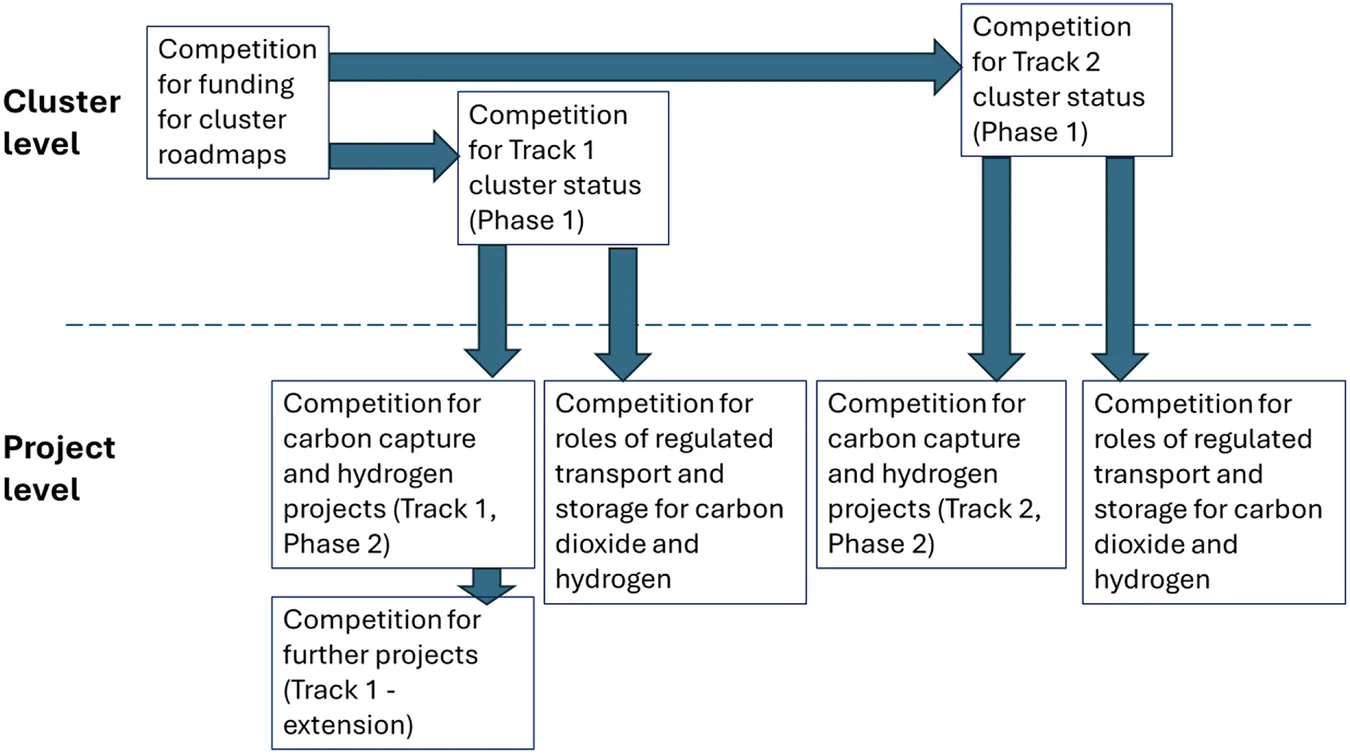
Industrial decarbonization cluster competitions in the United Kingdom. Source: authors. The diagram visualizes competitions at the geographic cluster level, e.g. large spatial areas of industrial carbon emissions, as well as across and within the specific project level, e.g. where specific interventions are being piloted and tested.

A distinctive feature of UK ID strategy is the cluster approach. Analysis showed that six industrial areas—Grangemouth in Scotland, Teesside and Humber in north east England, Merseyside in north west England, South Wales, and Southampton—were responsible for around half of the UK’s industrial emissions. Which clusters would receive resources first was decided on through a series of competitions. In 2019, companies and organisations in all clusters were invited to compete for a share of £1 million of funding to develop outline cluster decarbonisation roadmaps. These provided the basis for consortia to enter a two-stage ‘cluster sequencing’ competition to receive the main business model support for CCS and hydrogen (Fig. 2). In the first stage (known as Phase 1), clusters bid to be selected as the location for CCS and hydrogen transport and storage infrastructure, while in the second stage (Phase 2), specific carbon capture and hydrogen production projects within winning clusters were selected.

The winners of the first round of the cluster competitions were the HyNet NW consortium in the Merseyside cluster and an ‘East Coast’ consortium formed by the Humber and Teesside clusters^70^. The Scottish cluster was selected as a ‘reserve cluster’ in case one of the selected clusters dropped out. The competition for carbon capture projects (including blue hydrogen production) in the selected clusters then followed, with eight projects being confirmed in spring 2023 and reaching final investment decision in late 2024/early 2025. A second Phase 2 round competition for further CCS projects in Track 1 clusters (“Track 1-expansion”) was also held. A competition for the (green) hydrogen business models followed a little later, with 11 projects shortlisted in December 2023 and a further 27 shortlisted projects in early 2025. A Track 2 cluster competition was launched in 2023. This brought in Scotland through selection of the Acorn CCS pipeline project near Peterhead as well as the Viking project in Humber.

### Actors: distributing policy costs and benefits across industry, taxpayers and consumers

Based on our deductive coding of the qualitative data mentioned, the UK experience reveals three distinct arenas of political economy involving actors, technology, and geography.

Although ID policy in the UK began to emerge only from 2017 onwards, it was layered over competitiveness support policy for EIIs going back to the 2010s. Initially, this competitiveness policy did not have high political salience and was characterised mainly by a struggle between the Treasury and the EII lobby (with some sympathy for the latter from within business and trade departments).

In their messaging, the EII firms and associations have emphasised high energy costs relative to competitors in the EU, Turkey and North Africa^71–73^. Departments of business and trade have expressed similar concerns, for example, in policy documents proposing increases in policy cost exemptions^74,75^ and compensation for indirect carbon pricing^76^. By contrast, the Treasury has tended to take a somewhat sceptical view about cost pressures facing UK EIIs (Interview R118). For example, in its 2021 review of Net Zero, it urged caution about taxpayer-funded support to industry to offset the costs of climate policy, implying that the evidence base for such support was not robust enough (HM Treasury 2021: 37).

The relatively low political salience of EII competitiveness in this period meant that, while the UK did provide some support on energy costs, this was reactive in nature, lagging behind policy in other countries, while also being less generous. For example, an original decision to exempt some EIIs from policy costs on electricity was taken in 2012 and the scheme was launched in 2016, but this was less generous than those in competitor countries such as Germany in both depth and scope. Exemption schemes in Germany covered around 2000 firms but those in the UK covered only around 300. By comparison, only 5 out of 85 ceramic manufacturers in the UK are eligible for exemption from renewables costs and none get compensation for the emissions trading scheme and carbon price support policies. The value of exemptions for EIIs from renewables policy costs in the UK were worth around £300-400 million a year in the second half of the 2010s, but those in Germany were worth around €5 billion a year over the same period^77^. Overall, the regulator estimated that during 2016–2020 British EIIs faced electricity prices that were 40–50% higher than competitors in France, Germany and the Netherlands, after taking into account all exemptions and other forms of support^78^.

This situation began to change in the mid-2010s. The Paris Agreement revitalised the climate agenda and led to the adoption of net zero commitments; in the case of the UK in 2019. EII lobbying continued, but arguments for competitiveness support become intertwined with the decarbonisation agenda^79^. EII ‘sunset’ industry lobbies, those set to ‘lose’ or facing phase-out pressures, were joined by the ‘sunrise’ industries, those set to ‘win,’^80^ especially those in CCS and hydrogen. The objective was no longer just the protection of existing industries but support for new innovative, green industries that offered the prospect of capturing international markets. These factors placed greater pressure on the Treasury to support the ID policies being developed.

However, these factors were at work in all countries. What was particular to the UK was the enormous political shock of the Brexit referendum^81^. The vote was widely seen as a message from lower skilled and less educated workers in deindustrialised regions that they had been neglected^82^. This pushed issues of regional inequality and social injustice to the top of the conservative political agenda. Bringing about an industrial renaissance became a priority, with Theresa May making it a central focus of her administration, leading to the 2017 *Industrial Strategy* white paper.

The political significance of this agenda intensified further during the 2019 election, as a set of so-called ‘Red Wall’ Parliamentary seats in the north-west and north-east of England, previously seen as safe Labour seats became marginal and were won by the Conservative Party. The new government doubled down on what it framed as the ‘levelling up’ agenda, creating a dedicated new government department to address it. It is worth noting that the *Industrial Decarbonisation Strategy* devotes a whole chapter to levelling up.

Brexit was also seen as a vehicle by which the UK hoped to reclaim its lost glory on the world stage, with investments in industrial decarbonization being framed as a way for the country to not only build back better from the Covid pandemic, but to also attain a competitive edge on the world stage, showcasing global political leadership, and recovering sunk costs on long-lived natural gas infrastructure^83^. Industrial revitalization and global competitiveness were also a factor in the UK’s hydrogen strategy, which began with a focus on domestic uses for industrial applications and household heating, but expanded later to include hydrogen exports to Europe and ambitions to become “a major hydrogen player” globally^84^. Brexit has also been viewed as more than merely a populist platform, and instead one focused on protectionism, on insulating the UK from global rules that could be detrimental to its interest^85^, or on modernizing outdated infrastructure, especially in adjacent sectors such as automotive manufacturing^86^.

At the same time, ideas about ID policy were being developed that would help reassure the Treasury about cost. A CCS lobby convinced the government that a new approach to support based on mitigating the full value chain risk would be more successful than the previous abandoned demonstration projects. BEIS proposed developing CCS and hydrogen through the use of ‘contracts-for-difference’ (CfDs), which limited the exposure of the government to subsidy and had successfully demonstrated that learning-by-doing could reduce the costs of offshore wind over the 2010s. This in turn gave the Treasury the confidence to start thinking in terms of longer-term frameworks for support. According to a senior political figure instrumental in developing CCS policy:

*the Treasury was always very leery about committing lots of money to unproven technologies, that would take a very long time to pay off…So at the same time as promoting…carbon capture and storage, we were really pushing the acceleration of the offshore wind thing. And we actually got the Treasury to commit to this long term, 10-year cycle reduction, volume targets options, which again, was breaking all the orthodoxy.* (Interview R120).

These factors created what several interviewees saw as momentum for ID policy (Interviews R07, R12, R43, R48, R39), leading to high-level long-term commitments to resources matching those elsewhere in Europe. The Chancellor announced a headline figure of £20 billion for CCS over 20 years in 2023, and in 2024, the new Labour government expanded this pledge to £21.7 billion over 25 years^87^. This is equivalent to almost £870 million a year, while that for the hydrogen business models may be a similar magnitude by the 2030 s. The majority of this support will be borne by consumers through levies^88^. For comparison, Germany, with an economy roughly twice the size of the UK’s and a larger EII sector, has pledged roughly €3.3 billion a year to ID over the next 15 years^89^.

However, institutional factors, including the scepticism of the Treasury, did not disappear. Industrial strategy continued to show signs of instability. The 2017 strategy was quickly abandoned. Ad hoc decisions on bailouts for companies to avoid the closure of specific plants also continued to be taken, with the iron and steel sector providing some of the best-known examples^90,91^. Perceptions of instability fed uncertainty about the credibility of government commitment to ID (Interviews R12, R22, R54). Some in industry were also sceptical that commitments would be enough to reach targets:

*I think there’s…a lot of ambition and rhetoric, for example, in the hydrogen strategy that’s not been met. You know, the 5 GW target…but they’ve only actually committed funding for 1.5 GW so far by 2027. I don’t suddenly see them…allocating sufficient funding for another 3.5 GW of hydrogen from 2027 to 2030*. (Interview R21).

Treasury influence could also be seen in the relative complexity of ID policy in the UK^92^. A concern about value for money has meant that resources are disbursed through competitions and released sequentially over time. This was frustrating for some industry participants:

*I think it’s probably to reassure Treasury or other policymakers that the funding will be spread over the period…But that’s not a reason to then spend years coming up with a complicated policy mechanism for spacing those projects out…the cluster sequencing is completely unnecessary and counterproductive* (Interview R04).

Some industry interviewees contrasted the complexity of CfDs tied to carbon and natural gas prices in UK policy unfavourably with what were seen as a simpler approach in US policy:

*In contrast…instead of having a series of policies, they’ve got one single incentive. It’s the 45Q tax credit. If you can stop emitting carbon or avoid emitting carbon dioxide you will get a $50 a tonne grant, period*. (Interview R50).

### Technologies: lobbyists and advocacy coalitions for decarbonization solutions

The focus on CCS and hydrogen in UK policy has been justified on the basis that modelling showed that ID could not be achieved without these options, and that as immature technologies they need deployment support to achieve cost reductions. However, modelling (including for the *Industrial Decarbonisation Strategy* itself) also suggested that end-use efficiency improvements and electrification would be required^93,94^. At the same time, end-use efficiency was an underdeveloped approach, and electrification, while technologically mature, faced multiple barriers. Yet both of these have remained relatively orphan areas in ID policy.

It is therefore plausible that the interpretation of modelling results has been influenced by perceptions. In conditions of uncertainty, expectations about solutions are likely to be influenced by ideas promoted by advocacy coalitions. In this section we assess the different coalitions in the UK, and find that strong CCS and hydrogen coalitions contrast with much weaker electrification and circular economy coalitions.

CCS exists as a decarbonisation option in the UK because of storage capacity in depleted oil and gas fields^95^. But the nature of the CCS coalition is due not to that geological capacity *per se* but rather to the sector associated with it, i.e., an oil and gas industry that can see a commercial opportunity in those depleted fields, as well as soft assets in terms of competences and capacities to build and operate large-scale infrastructures, pipelines and pumps. The competencies and skills held by oil and gas companies could even act as a de facto monopoly, making it extremely hard to operate CO2 and hydrogen infrastructure without them.

Hydrogen has benefitted from the stronger bargaining and lobbying power of the Confederation of British Industry (CBI) and UK Steel, along with over 120 paid hydrogen lobbyists operating in Parliament, from multinational oil companies like Shell and BP to smaller hydrogen companies like Ryze. CCS has benefitted from a supportive Carbon Capture and Storage Association (CCSA) and venture firms interested in new innovations that would depend on CCS such as Bioenergy with Carbon Capture and Storage and Direct Air Capture with Carbon Storage^96^. These supportive coalitions have been successful in framing CCS and hydrogen fuel switching as not only technologically feasible, but minimally disruptive for both oil refineries and petrochemical plants, aligning them with the interest of incumbents^97,98^. CCS and hydrogen are also seen by many industry actors as types of end-use efficiency improvements and electrification options because those technologies are considered mature and do not require strong state intervention to de-risk investments, especially under the UK’s carbon contracts for difference approach^99^.

The CCS coalition has been coordinated since the early 2000s by the CCSA and comprises not only the oil and gas majors but also potential users such as electricity generators as well as specialist capture companies, technical experts, sympathetic government officials and MPs, the Climate Change Committee, some environmental NGOs and trade unions. It also involves regional actors, most notably the Teesside Collective, created by industrial interests in the north-east of England^100^. This coalition played an important role in bringing CCS back as a key part of UK decarbonisation policy^101^, proposing a new whole chain approach to policy support to meet the concerns of the Treasury about cost, and promoting the idea that CCS would be central to the ID, through both industrial capture and blue hydrogen^102^.

There is an increasing overlap between the CCS and hydrogen coalitions, with the oil and gas industry having interests in blue hydrogen as a potential solution to the risk of stranded assets^103^. The industry has also invested directly in hydrogen demonstration projects through the Oil and Gas Climate Initiative. Historically, the hydrogen lobby was organised through the UK Hydrogen and Fuel Cell Association, but in 2021 the oil and gas majors set up a new group, the Hydrogen Taskforce (later becoming Hydrogen UK) to feed into the design of the *Hydrogen Strategy*. The lobby is seen as highly effective^104^:

*…there’s a really sophisticated lobbying machine. There were hydrogen zones at the conferences, the party conferences. There’s a concerted effort…to upskill and warm up the next generation of MPs to the fact that hydrogen will be part of the solution.* (Interview R98; similar comments in Interview R81 and R16).

Both CCS and hydrogen coalitions have had opportunities to influence policy through various policy development working groups created by the government.

Because it has yet to be deployed at scale, CCS has attracted relatively little wider public attention (Interview R65). However, with the policy success of the lobby, there is growing opposition from some environmentalists and climate^105,106^. This is a concern amongst advocates:

*…I was the only person in this [climate] march of 10,000 people…with this placard that says “invest in CCS because it will halve the cost of decarbonisation,” I got so much abuse from the environmentalists…they hate it…*(Interviewee R122; similar points in Interviews R84, R86, R90).

Electrification has a lobby as well. Electricity already made up 32% of energy consumption for all industry in the UK in 2021, and the CCC expects this could rise to around half by 2040^107^. However, there are a number of barriers, including high prices and network charges (see above) and long queues for connection to the grid^108–110^.

Some of the ID cluster organisations are arguing for greater attention and support to electrification^111^. The business advocacy organisation the Aldersgate Group was one of the few actors pressing for a greater role for electrification. However, the industry body, Energy UK, only started working on the role of power sector on ID in 2024, and Electrify Britain focuses almost entirely on household energy use, not industrial use. Thus, the voices calling for a greater role for industrial electrification have remained uncoordinated and isolated, and policy attention has lagged. The government opened a call for evidence into electrification for ID only in 2023^112^.

Electricity is an incumbent technology with large corporate interests behind it, so the weakness of the lobby is somewhat surprising. There are a number of potential reasons for this. One is that, precisely because of the maturity of the technology, electricity industry actors do not perceive the need for active advocacy. A second is that generating and network companies are focused on the increasing demand for electricity from surface transport and residential heat, which is expected to be far larger than that from industry.

A final important coalition is the end-use material efficiency lobby. As noted above, the technical potential of end-use material efficiency remains largely unrealised, with multiple barriers to alternative design and circular economy approaches^113,114^. The complexity of the agenda (Interview R122) and a lack of data^115^ imply radical changes to business models and significant government support are needed.

But end-use efficiency and reduction have attracted relatively little attention^116,117^. The agenda has also suffered historically from being split institutionally between DESNZ and DEFRA (Interview R122). Only from late 2023 were a set of scoping exercises looking at the potential for end use efficiency and substitution published by government.

A coherent lobby for reducing industrial emissions through material efficiency improvements on the demand side in the UK does not exist as such^118^. There is a coalition for circular economy that involves a number or professional organisations and think-tanks, with the NGO Green Alliance playing a convening role^119^. There is also a considerable amount of research work on circular economy, coordinated through a Circular Economy Hub. The construction sector has an active network through the UK Green Building Council and the Construction Leadership Council (CLC) which runs projects looking at recycling and reuse for steel and cement. However, its work is largely technical and advisory. In electronics, there are a number of small NGOs and the consumer group Which, but these are focused on consumer behaviour change more than policy transformation. Overall, lobbying power even in these sectors is still weak.

### Geography: the spatial politics of ID resource distribution

ID policy in the UK has spatial elements as well, a dynamic we term “spatial politics” because it touches on issues of scale, clustering, and spatial agglomeration^120–123^. The cluster approach in UK ID policy appears to have been driven by the view that ID needed the deployment of CCS and hydrogen at scale, and that this in turn required locally shared, networked infrastructure. In the words of an official in BEIS: *The modelling said you need hydrogen and CCS, and that led to clusters* (Interview R123).

However, while concentrating policy resources on clusters has a logic of effectiveness and efficiency, it also has other consequences. One arises from the fact that cluster support is being allocated sequentially through a series of competitions, a choice driven by Treasury concerns about value for money. The clusters have different geological possibilities for hydrogen and carbon dioxide storage, types of industry, port facilities and administrative arrangements (Interviews R01, R29, R75, R95, R100, R104), all of which influence outcomes. Because a key criterion was geological storage options for CCS, the competitions worked against South Wales^124^. They have also tended to favour clusters with larger actors or established coalitions who are better placed to win, which in turn can reinforce these inequalities. For example, several interviewees noted that Teesside benefitted from a long history of coordination through the Teesside Collective (Interviews R13, R27, R37, R66). While policy makers recognised these differences and tried to take them into account (Interview R33), many actors felt they still had an effect.

A more fundamental issue is that a competitions approach involves making a choice about which clusters to support first and which to exclude. The dilemmas involved were well captured by industrialist Ian Temperton^125^ in 2018:

“The politics of the selection decision are a nightmare…All [clusters] present an excellent case for regeneration and maintenance of industrial skills…So if the government wants to give the steel industry an edge in a low carbon world it should pick Humber and South Wales…; if it thinks that chemicals are the most enduring part of the fossil fuel industry in a decarbonising world then it should pick two of Teesside, Scotland and Merseyside; if it is minded to promote industry in the devolved nations…it should pick Scotland and South Wales…What if they pick one of the two obvious steel locations and not the other? Or one of the chemicals hubs, say Scotland, and not the others? Won’t new investment in either existing or new facilities be naturally attracted to the region which has the CCS infrastructure and hence the ability to guarantee low carbon production.”

One dimension of these dilemmas is about industries, because these vary in how they are distributed. For example, choosing the Hynet NW and East Coast clusters in Track 1 has meant that well over half of the UK’s refineries have been excluded from early policy support. One of these, the Ineos refinery in Grangemouth, was even closed in April 2025 and converted into an import terminal for finished fuels, resulting in hundreds of job losses.

The cluster approach also concentrates policy resources in clusters as opposed to dispersed sites but due to historical factors a higher proportion of some industries are located in clusters than others (Table 3). It is noteworthy that cement, one of the larger emitters, is largely outside the coastal clusters as it developed near limestone sites, many in the centre of England. Similarly, metal processing is largely outside the coastal clusters, much of it in the West Midlands. The government did set up a competitionee in 2023 for decarbonisation plans in dispersed sites, but this was early-stage funding for only £5 million.

**Table 3:** Sectoral distribution of industrial carbon emissions in the United Kingdom

Industry	Total emissions (2018) (MtCO_2_e)	Percentage in clusters
Non-ferrous metals	0.9	3%
Paper	2.3	35%
Cement	6.8	38%
Glass	2.1	50%
Refining	13.1	83%
Primary iron production	11.1	100%

Calculated from Element Energy (2020) and Rattle et al. (2024). Note – sites in clusters are defined as located no more than 25 km (radial distance) from a major UK industrial cluster point.

A second dimension is the regional distribution of resources. Deindustrialisation in the UK has been particularly highly concentrated geographically^126^. As a result, the UK is one of the most regionally unequal countries in the OECD^127^. Policy to address these inequalities, including recent ‘levelling up’ initiatives, has largely failed^128^.

Offering some regions policy resources while excluding others has created tensions. These have arguably been greatest in Scotland, amplified by the politics of devolution and differences in the ruling parties between Westminster and Holyrood (Interview R86). When the decision was announced that Track 1 resources were to be focused on East Coast and Hynet NW clusters in January 2022, there was a strong response from the Scottish Energy Secretary:

“The UK Government’s decision not to award the Scottish Cluster clear and definitive Track-1 status is a serious mistake. Delaying or halting the deployment of the Scottish Cluster has serious consequences, including jeopardising the ID of Scotland and our just transition to net zero, creating an un-level playing field across the UK…”

The Acorn CCS project near Peterhead was selected to be in the Track 2 process, but the absence of funding flowing to Scotland was still attracting criticism in late 2024^129^.

The concern amongst industry interviewees was that decisions would disadvantage some regions, and lead to firms relocating:

*So is there a danger that there are some locations that are geographically disadvantaged to access to this and that as CO*_*2*_ *taxes wind up, through no fault of their own, they get kind of left behind and we end up with areas where you can’t do business of certain natures and that changes the profile of the country and leaves some folk disadvantaged?* (Interview R100; similar concerns in Interviews R68, R108, R76).

*And the playing field is now sharply biased in favour of Track One clusters. If you’re an industrial, enterprise, global anywhere in the world, and you’re thinking of…the UK, for whatever reason, where you’re gonna go?* (Interview R124; similar concerns in Interview R16).

## Discussion

**Fig. 3:**
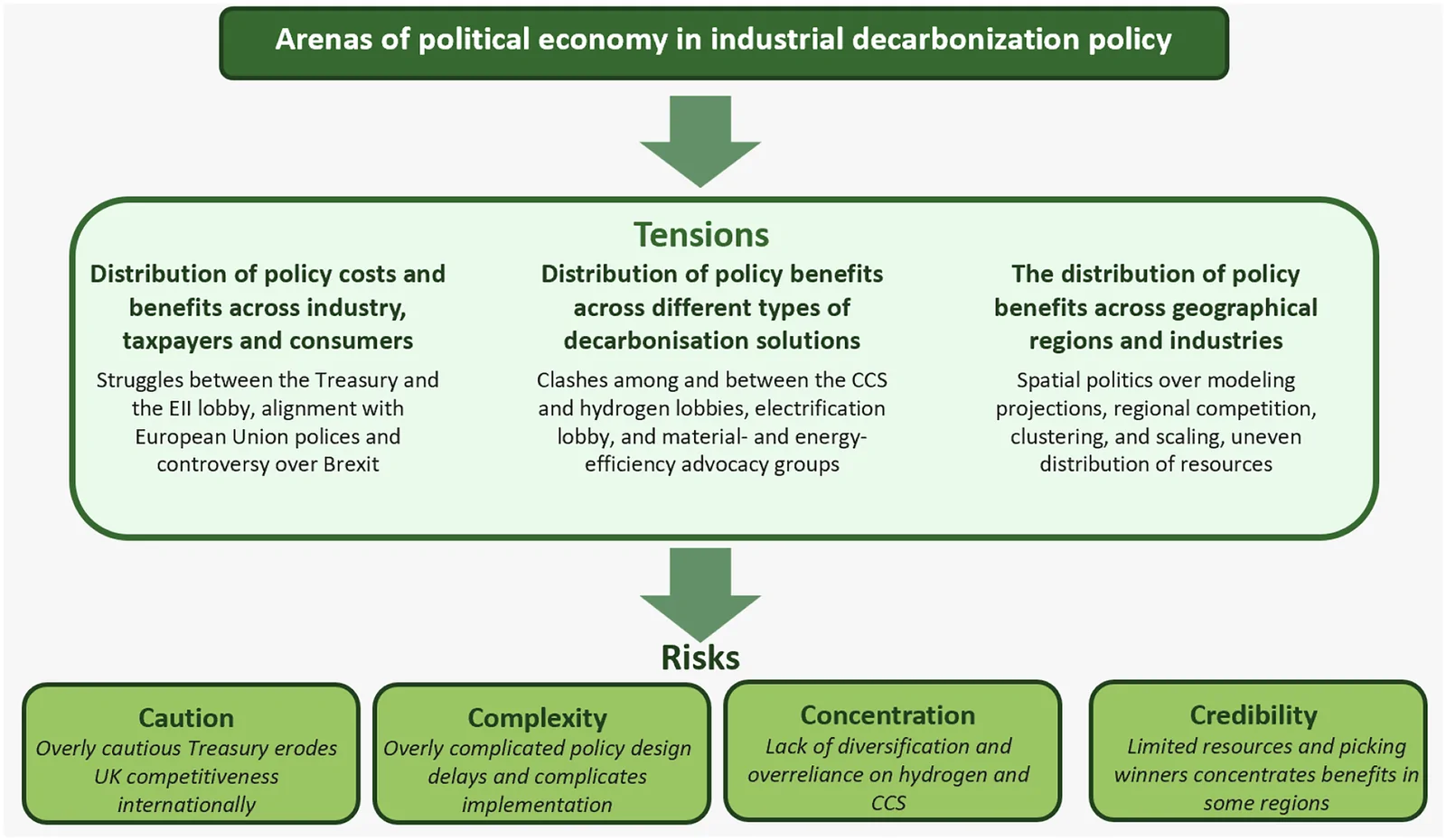
Visualizing the arenas of political economy for industrial decarbonization in the United Kingdom. Source: authors. The diagram illustrates how political economy ‘arenas’ arise over tensions related to the distribution of costs among actors, preferences over technological pathways, and spatial politics and the distribution of resources across regions. This culminates in risks related to caution, complexity, concentration and credibility.

In this paper, we have developed a framework for understanding the arenas of political economy related to industrial decarbonisation (ID) policy in terms of a set of potential conflicts over (1) the overall level of policy resources to be committed (2) the allocation of resources across decarbonising technologies or solutions, and (3) the allocation of resources across industries and regions (see Fig. 3).

In theory, fossil-fuel coalitions will seek new forms of innovation and competitiveness through decarbonization policy. Kupzok and Nahm^24^ conclude that this makes them receptive to a bargain: agreeing to meet climate goals in exchange for policies that support their decarbonization, especially fiscal policies that partially fund or de-risk their business transitions. In practice, however, our findings suggest there are a number of risks for UK ID policy arising from these arenas and their dynamics which slow or even stymie decarbonization at various levels. As depicted in Fig. 3, these are also related to four distinct risks that relate to caution, complexity, concentration and credibility.

One risk is that excessive caution on the part of Treasury continues to drive relatively weak protection from high energy costs, meaning that EIIs may be lost before they can be decarbonised, illustrated by the closure of the Grangemouth refinery and the loss of primary steelmaking in Port Talbot. Especially in steel and chemicals there are massive global overcapacities and structural disadvantages regarding energy and feedstock prices compared to other world regions. It might simply not be possible to subsidize them into competitiveness in the long term.

Moreover, if Treasury concerns about over spending reassert themselves over contingent politics, this may lead to reductions in support for ID in future and the risk that UK EIIs will not be able to compete in a world where low-carbon industry is required. Another force or tension weakening the UK’s capabilities for decarbonization is the tendency for large scale investments in low carbon sectors to be abandoned directly with a focus instead on ‘greening’ financial expertise located in the City of London, thereby making green finance the centrepiece of British industrial policy, not technology^130^.

It is already the case that the complexity of policy design has meant that the development of detailed delivery instruments has been slower than originally anticipated, and progress towards targets is slipping. In late 2024, the government admitted that the original CCS targets for 2030 would not be met, and the first CCS plant will not be operating until 2028 at the earliest, rather than the mid-2020s. Similarly, even if all the shortlisted hydrogen projects progress, they will fall far short of the target of 5 GW of electrolytic hydrogen by 2030. In fact, of the first round of projects signalled in December 2023, only three projects had progressed to final investment decision a year later.

Beyond overall levels of support, there is a risk that ID policy may be distorted, overly focused on CCS and hydrogen due to the relative strength of their lobbies, while electrification and demand-side solutions are underdeveloped. The danger of over-reliance on CCS and hydrogen approaches is that they may ultimately turn out to be more expensive than others, are not scalable enough to realistically deliver targets on time^131,132^, or cannot reduce emissions sufficiently^133,134^. Further risks arise from the idea that the UK can become a leader in CCS and hydrogen technologies. Global markets for these technologies may be smaller than anticipated, especially if they turn out to be more costly than expected^135^. Finally, it is already the case that the prospect of CCS and blue hydrogen developments in particular has stimulated opposition amongst some civil society organisations and legislators, and there is a risk that this opposition will spread.

A different set of risks arise from the political economy of the allocation of policy resources across industries and regions. One is that those industries and regions not allocated resources early on will disengage with the policy. This may not be a problem if they develop their own alternatives—for example, the cement industry through the Peak Cluster project. However, if the concentration of support in certain regions leads to a loss of credibility in the ID project as a whole, this would be more damaging. One effect might be that new investment in EIIs will be drawn to Track 1 regions with CCS and hydrogen infrastructure, meaning that other regions lose out. A more extreme version is that existing firms in regions not receiving policy support may relocate, because they can decarbonise more easily in Track 1 or 2 clusters.

In conclusion, the path of industrialisation decarbonisation policy in the UK since the mid-2010s can be seen as shaped by dynamics in three arenas concerning the distribution of costs and benefits among different groups, sectors and technologies, and geographic regions and scales. One concern is the ascendancy of contingent politics driven by concerns over climate change and regional inequality over institutional factors, leading to a weakening of what is seen as the power of Treasury orthodoxy, and commitments to significant ID policy resources at a high level. In particular, the Brexit vote revealed new political cleavages meaning that the focus of political competition moved away from costs placed on consumers and taxpayers towards action to reduce regional inequalities and revive manufacturing^136^. Second, strong, inter-related CCS and hydrogen lobbies have reinforced a view in government that support for the development of these technologies is a priority, and in turn that they must be deployed in clusters. Finally, Treasury concerns about value for money have created extreme competition and tensions in industry and between regions and central government.

The arenas, dynamics and risks identified here are specific to the UK case. That means some elements of our findings are not generalizable in the sense that the UK economy is much smaller than the larger and more diverse economies of China and the United States; the strong legal foundations offered by the UK’s Climate Change Act; the history of the UK having gas economy with an abundance carbon storage capacity in both depleted oil and gas reservoirs and salt caverns; and a readily available national supply of natural gas which can be used to manufacture hydrogen^15^. Policymakers around the world, however, can replicate other aspects of the UK approach, such as strong political commitment for ID policy across the political divide of conservatives, liberals, the green party and labour party; fairly consistent and predictable climate policy and binding carbon targets; the importance of financing ID with public resources and stimulating decarbonization with public procurement; and reliance on harbours, seaports, and pipelines to distribute hydrogen and transport CO2 to remote areas and vital industrial clusters^15^.

However, our approach to the political economy of ID, one that highlights potential conflicts over the size of policy resources and its distribution amongst decarbonisation solutions, industries and regions, is much more widely applicable. Our arenas framework could help other regions, clusters, and cultures achieve a stronger understanding of the evolution of ID policies as they continue to develop across a range of contrasting contexts.

## Methods

### Research interviews

This study draws on original and secondary data sources. A key source is a set of interviews conducted with 125 participants in and observers of ID processes and policy, carried out over the period December 2021 to June 2023 (see Supplementary Table 1). These interviews provide a range of perspectives from industry, including trade associations, national government and policymakers, local authorities, start-up companies, NGOs, and academics. The interviews were semi-structured, enabling the research team to follow up different topics that emerged in a flexible way. Interviews lasted between 30 and 180 min, were recorded and then transcribed. To protect the anonymity of respondents, they were assigned a generic respondent number (R01, R02, etc.). There is a good representation of the major actors across the industrial clusters in the UK, but the largest single category of interviews (69) was with industry respondents. The transcribed text was then analysed manually using a deductive approach relating to the dimensions of political economy in the framework. Informed consent was gained from all respondents, and ethics approval was granted by the University of Sussex (Ref: ER/BS289/6).

### Document analysis and observations 

A second important data source was relevant documentation, including: policy documents related to the UK’s ID mission; Parliamentary reports, documents and online information produced by regulatory agencies and standards bodies, and publicly available reports from industry bodies, academics, NGOs, trade bodies, etc.

The study also draws on presentations and discussions at a number of meetings on ID or related topics, including: an All-Party Manufacturing Group roundtable on energy intensive industries in October 2021, ID Research and Innovation Centre (IDRIC) annual conferences in June 2022 and May 2023, IDRIC Policy Forum meetings in October 2022 and March 2023, and a roundtable on greening the Treasury in April 2024.

A key limitation of the case study is that it focuses on a specific time period, essentially from the late 2010s to the mid-2020s. This was primarily a period of policy development, in which the level of policy resources, their allocation to different decarbonising technologies and approaches, and their initial distribution across different regions and industries became clear. However, at the time of writing, implementation, in the sense of final investment decisions and construction on the ground, is only beginning.

## Supplementary information

Supplementary Information

## Data Availability

The datasets generated and/or analyzed during the current study are not publicly available due to privacy protections and ethics requirements.
