# Supplementary material for: Actors, technology and geography: arenas of political economy for industrial decarbonisation policy in the United Kingdom
Source: NPJ Environ Soc Sci. 2026 Nov 4;1(1):22. doi: 10.1038/s44432-026-00022-4 (PMC13736638; doi:10.1038/s44432-026-00022-4)
Supplement: Supplementary file 1 [file 44432_2026_22_MOESM1_ESM.pdf]

Supplementary Information

Supplementary Table 1

List of interviews

Code	Institution	Type	Date
R01	BEIS	Policy	10.02.22
R02	University of Manchester, the Tyndall Centre for Climate Change Research	Academia	16.02.22
R03	Equinor ASA	Industry	25.02.22
R04	Wood	Industry; provides technical and industry expertise	28.02.2022
R05	University of Oxford	Academia	2.03.22
R06	Centrica	Industry	3.03.22
R07	VPI Immingham	Industry	4.03.22
R08	ITM Power	Industry	4.03.22
R09	Phillips66	Industry	7.03.22
R10	SSE	Industry	10.03.22
R11	Triton Power	Industry	16.03.22
R12	Triton Power	Industry	16.03.22
R13	Humber LEP	Local authorities	17.03.22
R14	Centrica	Industry	18.03.22
R15	VPI Immingham	Industry	18.03.22
R16	Cadent	Industry	8.03.22
R17	University of Sussex	Academia	20.03.22
R18	University of Chester	Academia	21.03.22
R19	Progressive Energy	Industry	21.03.22
R20	University of Chester	Academia	21.03.22
R21	Progressive Energy	Industry	22.03.22
R22	Peel	Industry	22.03.22
R23	North West Business Leadership Team	Industry/Business	23.03.22

R24	BEIS (ET&CG, Industrial Energy)	Policy	25.03.22
R25	Phillips66	Industry	28.03.22
R26	Progressive Energy	Industry	29.03.22
R27	North Lincolnshire Council	Local authorities	4.04.22
R28	British Steel	Industry	12.04.22
R29	Imperial College London	Academia	12.04.22
R30	Element Energy	Industry	13.04.22
R31	Offshore Renewable Energy Catapult	Industry/Academia (research)	13.04.22
R32	BEIS	Policy	14.04.22
R33	BEIS	Policy	14.04.22
R34	Inovyn	Industry	15.04.22
R35	Pilkington	Industry	19.04.22
R36	Encirc	Industry	20.04.22
R37	National Grid	Industry	21.04.22
R38	Hull city council	Local authorities	22.04.22
R39	SSE	Industry	25.04.22
R40	SSE	Industry	25.04.22
R41	SSE	Industry	25.04.22
R42	Mitsubishi Power Europe	Industry	27.04.22
R43	EssarOil	Industry	29.04.22
R44	University of Sheffield, UK CCS Research Centre	Academia	1.04.22
R45	Norwegian British Chamber of Commerce	Industry	26.05.22
R46	Drax	Industry	13.06.22
R47	Manchester University	Academia	17.09.22
R48	NECCUS	Industry: trade group	22.09.22
R49	NECCUS	Industry: trade group	22.09.22
R50	Energy Transition Advisor Limited, Storegga	Industry	06.10.22

R51	Herriot-Watt University	Academia	05.10.22
R52	Kellas Midstream	Industry	05.10.22
R53	British Geological Survey (BGS)	Policy-Research-Academia	10.10.22
R54	Lanzatech	Industry	10.10.22
R55	Stanford University	Academia	05.10.22
R56	North Sea Transition Authority	Policy	14.10.22
R57	Wales West Utilities	Industry	21.0.22
R58	SBH4	Industry	24.10.22
R59	ECITB	Trade	24.10.22
R60	Carbon8	Industry	24.10.22
R61	Aker Solutions	Industry	25.10.22
R62	TuvSud	Policy-Research	25.10.22
R63	UCL	Academia	28.10.22
R64	British Geological Survey (BGS)	Policy-Research	27.10.22
R65	Imperial College London, Just Transition Scotland	Academia	26.10.22
R66	SWIC, Industry Wales, Baxter360	Industry	27.10.22
R67	Wales Hydrogen Centre, Uni. of South Wales	Academia	28.10.22
R68	CT Energy, Southeast Water UK, CPH2, Nuclear Decommissioning Authority, Cadent Gas Ltd.	Industry	27.10.22
R69	Tarmac	Industry	28.10.22
R70	University of Strathclyde	Academia	28.10.22
R71	University of Strathclyde	Academia	31.10.22
R72	Plansea	Industry	31.10.22
R73	Manchester Uni.	Academia	31.10.22
R74	Aker Solutions	Industry	24.10.22
R75	Carbon Trust	Policy	03.11.22
R76	Storegga	Industry	03.11.22

R77	Renewables hydrogen alliance	Policy	09.11.22
R78	Just Transitions Committee, Uni. of Bath	Academia	09.11.22
R79	Airhive	Industry	10.11.22
R80	Engaging Next Gen. Industry, Fuel Change	Trade	10.11.22
R81	University of Aberdeen	Academia	28.10.22
R82	Reliagen, Association of Renewable Energy and Clean Technology, BioSci	Industry	10.11.22
R83	Suez Group	Industry	11.11.22
R84	Shell	Industry	11.11.22
R85	Green Alliance	Policy	09.11.22
R86	SCCS	Policy	14.11.22
R87	Vale	Industry	31.01.23
R88	Storegga (Pale Blue Dot)	Industry	14.11.22
R89	Tees Valley CA, CER Technologies, Ltd.	Local authorities	08.11.22
R90	ENSUS UK Ltd., NEPIC	Trade	15.11.22
R91	Innovate UK (Enterprise Services at Heriot-Watt)	Policy-Research	16.11.22
R92	VNZS, VItaBio Group	Industry	07.11.22
R93	Oxford	Academia	16.11.22
R94	British Geological Survey (BGS)	Policy-Research	18.11.22
R95	Edinburgh University	Academia	18.11.22
R96	Honeywell	Industry	18.11.22
R97	Sheffield University	Academia	18.11.22
R98	BEIS (NZBI - Clean Heat)	Policy	15.11.22
R99	Low Carbon Contracts Company	Policy-Industry	24.11.22
R100	Shell	Industry	25.11.22
R101	Intercontinental Energy, Everything About Hydrogen, Hydrogen Council, Sustainable Energy Council	Industry	25.11.22

R102	Scottish Parliament	Policy	02.12.22
R103	British Geological Survey	Academic/Research/Industry	06.12.22
R104	Durham University	Academia	09.12.22
R105	retired British Steel	Industry	16.12.22
R106	Helmholtz Centre, RIFS-Potsdam	Academia	
R107	BEIS	Policy	07.12.22
R108	Kellas Midstream (NEPIC)	Industry	22.12.22
R109	Sembcorp Energy UK (Wilton Int.)	Industry	11.01.23
R110	RWE	Industry	15.12.22
R111	Protium Green Solutions, Everything About Hydrogen (UK Hydrogen & Fuel Cell Association)	Industry	19.01.23
R112	Inovyn	Industry	11.05.23
R113	Cadent	Industry	17.05.23
R114	Mineral Products Association	Industry	21.06.23
R115	Institution of Gas Engineers and Managers (IGEM)	Industry	23.06.23
R116	Storegga	Industry	27.06.23
R117	Environment Agency	Policy - Regulator	27.06.23
R118	Energy Intensive Users Group	Industry	26.10.22
R119	Former civil servant	Policy	03.03.22
R120	Responsible Steel	Industry	02.12.22
R121	Imperial College	Academia	21.10.22
R122	Green Alliance	Policy	15.11.22
R123	BEIS	Policy	16.12.21
R124	Camirus	Research/Policy	10.09.22
R125	University of South Wales	Academia	10.01.23
